# Impact of *ERG6* Gene Deletion on Membrane Composition and Properties in the Pathogenic Yeast *Candida glabrata*

**DOI:** 10.1007/s12013-024-01599-w

**Published:** 2024-10-31

**Authors:** J. Jacko, M. Morvová, N. Tóth Hervay, D. Eliaš, Y. Gbelská, I. Waczulíková, D. Gášková, M. Balážová, L. Šikurová

**Affiliations:** 1 Department of Nuclear Physics and Biophysics, Faculty of Mathematics, Physics and Informatics, Comenius University Bratislava, Bratislava, Slovakia; 2 Department of Microbiology and Virology, Faculty of Natural Sciences, Comenius University Bratislava, Bratislava, Slovakia; 3 Institute of Physics, Charles University, Prague, Czechia; 4 Centre for Biosciences SAS, Institute of Biochemistry and Genetics of Animals SAS, Bratislava, Slovakia

**Keywords:** *Candida glabrata*, *ERG6*, Eburicol, Ergosterol, Phospholipids, Transmembrane potential

## Abstract

The *ERG6* gene is crucial for the biosynthesis of ergosterol, a key component of yeast cell membranes. Our study examines the impact of *ERG6* gene deletion on the membrane composition and physicochemical properties of the pathogenic yeast *Candida glabrata*. Specifically, we investigated changes in selected sterol content, phospholipid composition, transmembrane potential, and *PDR16* gene activity. Sterol levels were measured using high-performance liquid chromatography, the phospholipid profile was analysed via thin-layer chromatography, transmembrane potential was assessed with fluorescence spectroscopy, and gene expression levels were determined by quantitative PCR. Our findings revealed a depletion of ergosterol, increased zymosterol and eburicol content, an increased phosphatidylcholine and a reduced phosphatidylethanolamine content in the *Δerg6* strain compared to the *wt*. Additionally, the *Δerg6* strain exhibited membrane hyperpolarization without changes in *PDR16* expression. Furthermore, the *Δerg6* strain showed increased sensitivity to the antifungals myriocin and aureobasidine A. These results suggest that *ERG6* gene deletion leads to significant alterations in membrane composition and may activates an alternative ergosterol synthesis pathway in the *C. glabrata Δerg6* deletion mutant.

## Introduction

In recent decades, biomedical research has shown a growing interest in the yeast *C. glabrata*. Originally considered a harmless part of the human microbiome [1], *C. glabrata* has gained significant attention due to the increasing incidence of invasive yeast infections, which lead to severe complications, particularly in immunocompromised patients [2]. *C. glabrata* is characterized by increased resistance to commonly used antifungals, which poses a major challenge in treating infections caused by this pathogen [3]. Most clinically used antifungals target the biosynthesis of (1–3)-β-D-glucan, an abundant compound of the yeast cell wall, or ergosterol in the cell membrane. Altered sterol production can affect the phospholipid content in cell membranes, typically changing their structure and fluidity [3]. These changes can subsequently lead to differences in transmembrane potential [4] in yeast as *Saccharomyces cerevisiae* and *Kluyveromyces lactis* [5]. However, no research has yet been conducted on this issue in the pathogenic yeast *C. glabrata*. One of the genes involved in ergosterol synthesis is *ERG6*, which encodes the enzyme C-24 methyltransferase. The dysfunction of the *ERG6* gene leads to the formation of cells that are viable but exhibit defects in natural growth, cell division, and resistance to stress conditions. With the increasing resistance to current antifungals in *C. glabrata* [6–8], finding new therapeutics or improving the efficacy of existing drugs may be a priority. Therefore, targeting specific cellular processes of the pathogen is a promising approach. Deletion of the *ERG6* gene disrupts ergosterol biosynthesis and cell integrity, leading to severe cellular defects and reduced viability of *Cryptococcus neoformans* yeast cells [9], which has yet to be verified for *C. glabrata*. Experiments with various yeast, such as *S. cerevisiae, K. lactis*, and *Candida albicans*, have highlighted the importance of ergosterol for membrane permeability, drug resistance, protein transport to the plasma membrane, sporulation, and endocytosis [10–18]. Further studies have shown that *ERG6* mutants of *C. albicans* are hypersensitive to sterol synthesis inhibitors but do not exhibit increased sensitivity to azole antifungals [19]. On the other hand, [20] documented that mutations in the *ERG6* gene increases the efficacy of polyenes against *C. albicans* and [21] demonstrated that deletion of the *ERG6* gene increases susceptibility to azoles. Similar results were observed in the yeast *Aspergillus fumigatus*, where deletion of the *ERG6* gene led to increased efficacy of azole antifungals and reduced cell viability [22]. The effect of *ERG6* gene deletion on the effectiveness of myriocin and aureobasidin A antifungals in *C. glabrata* has not yet been explored, but it is known that myriocin and aureobasidin A in synergy with fluconazole, enhance the effect of fluconazole in *C. glabrata* [23].

There is evidence suggesting that eburicol may perform similar functions to ergosterol in yeast cells [24]. Eburicol is a sterol whose role in yeast is relatively less explored compared to ergosterol. The levels of eburicol in *C. glabrata* have not yet been explored in terms of eburicol as a potential substrate for ergosterol synthesis.

Lipids are one of the four main macromolecules essential for cell function. Depending on their properties, lipids play many roles in the cell, including controlling membrane structure and fluidity, signalling, membrane-associated functions, and influencing virulence and resistance [25]. Phospholipids are the major structural components of cell membranes and are essential for vital cellular processes [26]. Membranes contain four main phospholipids, namely phosphatidylethanolamine (PE), phosphatidylcholine (PC), phosphatidylinositol (PI), and phosphatidylserine (PS) [27, 28]. In Almost all yeast membranes, PC and PE are present at 60–70% of total phospholipids [29]. Inositol phosphorylceramide (IPC) is an essential sphingolipid found in fungi, plants, and some protozoa [30]. Complex sphingolipids, such as IPC and sphingomyelin in mammals, are major components of the outer leaflet of the eukaryotic plasma membrane. These lipids, along with sterols, are thought to be involved in the formation of microdomains known as lipid rafts. These rafts have various functions, including transporting lipid-modified proteins and assembling and activating signal transduction complexes [31]. Given the non-mammalian nature of IPC synthase, this synthase is an attractive drug target. It has been approved as a target in pathogenic fungi and kinetoplastid protozoa.

The Pdr16p protein (encoded by *PDR16* gene) belongs to the Sec14 family of phosphatidylinositol transport proteins, which are designed to supply phosphoinositide to kinases that produce phosphatidylinositol phosphates. These lipid transport proteins thus regulate many events in membrane transport and signaling that require phosphatidylinositol phosphates [32]. The absence of Pdr16p leads to increased sensitivity to azole antifungals in several yeast species, including *C. albicans* [33, 34], *C. glabrata* [35], *S. cerevisiae* [36, 37] and *K. lactis* [38].

In our study, we will assess the effects of *ERG6* gene deletion on the content of selected lipids and phospholipids in the membranes of *C. glabrata* yeast and on the transmembrane potential, with the aim of gaining new insights into the molecular mechanisms affecting the biological properties of the pathogenic yeast *C. glabrata* and its resistance to antifungals. This will contribute to the understanding of the *ERG6* gene as a potential target for enhancing the efficacy of current antifungals or as a site for a new potential treatment approach.

## Material and Methods

### Yeast Strains and Growth Media

The *C. glabrata* strains used in this study were *Cglig4Δ lig4::HIS3 trp1* (hereafter referred to as *wild-type - wt*) and its isogenic derivate *Cglig4Δ erg6 lig4::HIS3 erg6::TRP1* (hereafter referred to as *Δerg6*). *C. glabrata* cells were cultivated in liquid YPD (yeast extract peptone dextrose) (1% yeast extract, 2% peptone, 2% glucose).

### Analysis of the Ergosterol, Zymosterol, and Eburicol in the Membranes

For the analysis of selected sterols of the *Candida glabrata* membranes, we first isolated the mixture of non-saponifiable lipids from 1 × 10^9^ cells/ml. The cells were broken by homogenization with glass beads and incubated in 3 ml of 60% KOH in 50% methanol for 2 h at 70 °C. Sterols were extracted from the mixture of non-saponifiable lipids using 3 ml of n-hexane (Honeywell, USA). Subsequently, the sterol extracts were dried under a stream of N_2_ and dissolved in 1 ml of 100% methanol (Honeywell, USA) to a concentration of 0.84 mmol/l. The prepared samples were analysed by Prominence 20 A modular HPLC (High-Performance Liquid Chromatography) system (Shimadzu, Japan) with an SPD-20A absorption detector. Spectra were recorded at wavelengths of 209 nm for eburicol, 282 nm for ergosterol and 211 nm for zymosterol. A mixture of methanol and redistilled water in a ratio of 95:5 was used as the mobile phase at a flow rate of 0.8 ml/min. Measurements were taken using a MacheryNagel® C18 column, 15 cm in length, with an internal diameter of 4.6 mm and a particle diameter of 5 µm. The amount of sterol in the sample was determined using calibration curves of HPLC standards. Sterol analyses were conducted at a temperature of 22 °C.

### Analysis of the Membrane Phospholipid Profile

#### Phospholipid extraction

Each sample contained 2 × 10^9^ cells/ml from which a cell homogenate was created. Lipids were extracted from the samples using 4 ml of a chloroform-methanol-HCl mixture (60:30:0.26) for 1 h at 30 °C while shaking. After addition of 4 ml MgCl_2_ (0.1 mol/l) the mixture was vortexed thoroughly and incubated for another 30 min. After separation of aqueous and organic phase by centrifugation (5 min, 1500 g) the organic phase was removed, and the lipids dried under a stream of N_2_.

#### Separation of phospholipids by thin layer chromatography

The extracted phospholipids were dissolved in 25 µl of a chloroform-methanol mixture (2:1) and applied to a silica gel plate. The plate was developed at 25 °C for 2 h in a chromatography tank, using a mobile phase mixture of chloroform-methanol-acetic acid-water (75:45:3:1). The separated phospholipids were visualized by staining with iodine vapors.

#### Quantitative determination of phospholipids

The silica gel plate with separated phospholipids was moistened with distilled water. Spots corresponding to individual phospholipids were scraped off the plate into sealable Pyrex tubes. Samples were dried for 30 min at 105 °C. After cooling, 200 µl of a sulfuric acid-chloric acid mixture (9:1) was added to the samples that were boiled in a sand bath for 30 min at 180 °C. After cooling, 4.8 ml of an ANSA (1-amino-2-naphthol-4-sulfonic acid) molybdate ((NH4)6Mo_7_O_24_ × 4 H_2_O) mixture was added and incubated for 30 min at 105 °C. After cooling, the vortexed samples were sedimented by centrifugation (3 min, 2500 g). 200 µl from each sample was transferred into a 96-well plate to measure absorbance at 830 nm. The amount of phosphate per mg of protein (µg Pi/mg protein) was determined based on a calibration curve of K_2_HPO_4_ (0, 0.4, 0.7, 1, 1.5, 2.5, and 4 µg). The amount of individual phospholipid was determined as the percentage of the given phospholipid relative to the total amount of phosphorus in the sample.

### TMP Value

For the quantification of the transmembrane potential (TMP) difference of the plasma membrane in *C. glabrata* strains, cells were grown in YPD to the exponential phase. They were then collected, washed twice with distilled water, and resuspended in a 10 mmol/l citrate phosphate buffer solution (pH 6.0) to an OD_600_ of 0.4. Next, 2 ml samples of the cell suspension were adjusted to an OD_600_ of 0.125 (i.e., 1.8 ×10^6^ cells/ml) and subsequently labelled with the potentiometric probe diS-C3[3] (3,3’-dipropylthiadicarbocyanine) (Sigma-Aldrich, USA) to a final probe concentration in the sample of 1.5 × 10^−7 ^mol/l. Fluorescent emission spectra of the cell suspensions were measured using a FLUOROMAX 4 spectrofluorometer (Jobin-Yvon, Japan). The excitation wavelength was 508 nm, and the fluorescence emission spectra were recorded in the range of 560–590 nm. The difference in the position of the fluorescence maxima (λ_max_) was determined as the difference between the maximum wavelength of emission at steady state and the maximum wavelength of emission at the beginning of the measurement according to [39]. TMP difference measurement was performed at a temperature of 25 °C.

### Susceptibility Assay

The susceptibility of *C. glabrata* strains to sphingolipid biosynthesis inhibitors was tested by spott assay. Myriocin (Sigma-Aldrich, USA, prepared in DMSO) and aureobasidin A (Sigma-Aldrich, USA, prepared in ethanol) were used. Briefly, overnight cultures grown in YPD medium (150 rpm, 30 °C) were diluted to a density of 1 × 10^7^ cells/ml. Tenfold serial dilutions were prepared and 5 μl aliquots of each dilution were spotted onto solid YPD plates, containing the indicated concentrations of tested drugs. The concentrations were as follows: myriocin 1; 1.5; 2; 2.5; 3 µg/ml, aureobasidin A 0.05; 0.07; 0.1; 0.2; 0.5 µg/ml. The plates were incubated at 30 °C for 2 days.

### Quantitative PCR

The relative levels of gene expressions were assessed by quantitative PCR (qPCR). Overnight cultures of *C. glabrata* strains grown in YPD medium (150 rpm, 30 °C) were resuspended in a fresh YPD medium to concentration of 5 × 10^6^ cells/ml and grown to mid-logarithmic phase. Total RNA was isolated by the GeneJET RNA Purification Kit (ThermoFisher Scientific, USA). Purity and integrity of isolated total RNA was assessed by spectrophotometry and agarose gel electrophoresis and further treated with DNase I, RNase-free (ThermoFisher Scientific, USA) to remove contaminating genomic DNA. First-strand cDNA was synthesized from 1 µg of treated RNA using Revert AidTM H Minus MMuLV Reverse Transcriptase (Thermo Fischer Scientific, USA). qPCR was prepared using the HOT FIREPol® EvaGreen® qPCR Mix Plus (ROX), 5x (Solis BioDyne, EU). Amplification was carried out in triplicates in the 7900 HT Fast Real-Time PCR System (Applied Biosystems, USA). Specificity of qPCR was verified by melting curves analysis after completion of qPCR cycles. The reporter signals were analysed using the ABI SDS 2.2.2 software (Applied Biosystems, USA). *ACT1* gene was used to normalize the levels of *PDR16* gene expression. The gene expression levels were determined by 2^–∆∆Ct^ method [40].

### Data Processing

All data were graphically processed using OriginPro 2018 software (Origin Lab, USA). The normality of the data distribution was verified using the Shapiro-Wilk test, which revealed no deviations from normality in the data distribution. To assess differences between the *wt* and *Δerg6* groups, we used a two-tailed F-test. The statistical significance of differences between the two populations was evaluated using an unpaired t-test. All statistical analyses were performed using Statsdirect software (Statsdirect, UK).

## Results

### Presence of Ergosterol, Zymosterol, and Eburicol in *C. glabrata* Membranes

**Fig. 1:**
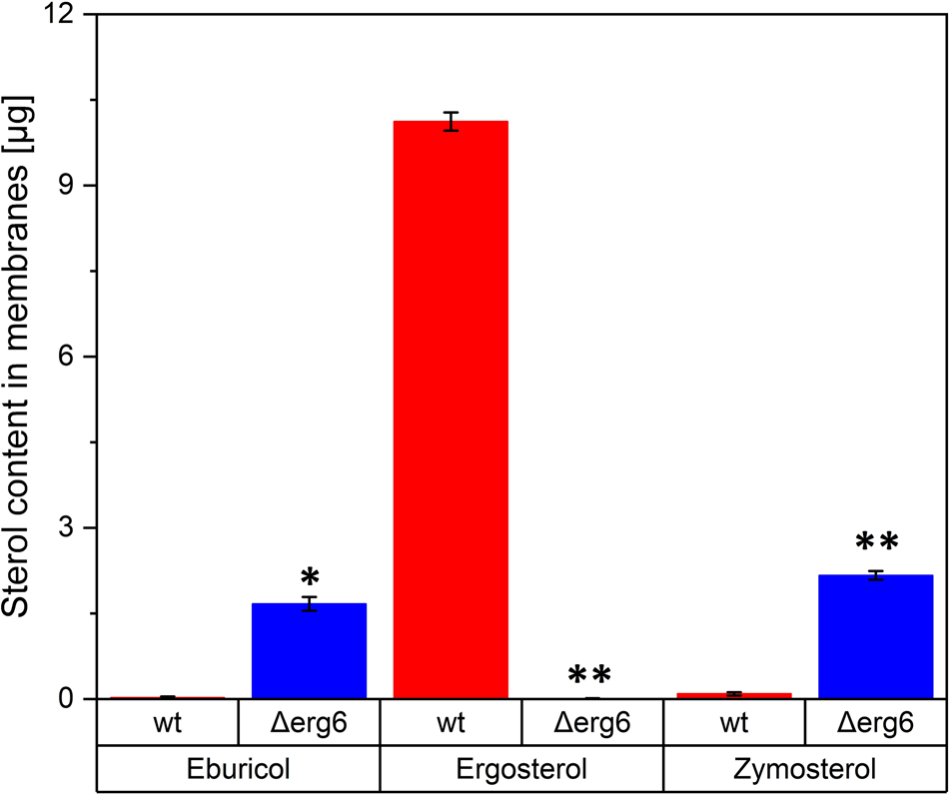
Content of selected sterols (eburicol, ergosterol, and zymosterol) in the membranes of *C. glabrata*. (*p < 0.025, **p < 0.0001; unpaired t-test *Δerg6* versus *wt*; results are expressed as mean values of three independent experiments ± standard deviations)

Using HPLC, we analysed the impact of *ERG6* gene deletion on the presence of ergosterol, eburicol, and zymosterol in the membranes of *C. glabrata* yeast. The levels of eburicol, ergosterol, and zymosterol in the *C. glabrata* membrane were quantified using calibration curves created by diluting HPLC standards of the respective sterols (Table 1). We observed an increase in the levels of eburicol and zymosterol, and a depletion of ergosterol in *Δerg6* compared to *wt* (Fig. 1).

**Table 1:** Eburicol, ergosterol and zymosterol content in the membranes of *C. glabrata* strains

Sterol	*wt [µg]*	*Δerg6 [µg]*	*p*-value *Δerg6* vs *wt*
Eburicol	0.025 ± 0.002	1.667 ± 0.012	p < 0.025
Ergosterol	10.120 ± 0.160	0.005 ± 0.001	p < 0.0001
Zymosterol	0.090 ± 0.003	2.166 ± 0.076	p < 0.0001

The results are expressed as mean values of three independent experiments ± standard deviation

### Phospholipid Profile of the *C. glabrata* Membranes

**Fig. 2:**
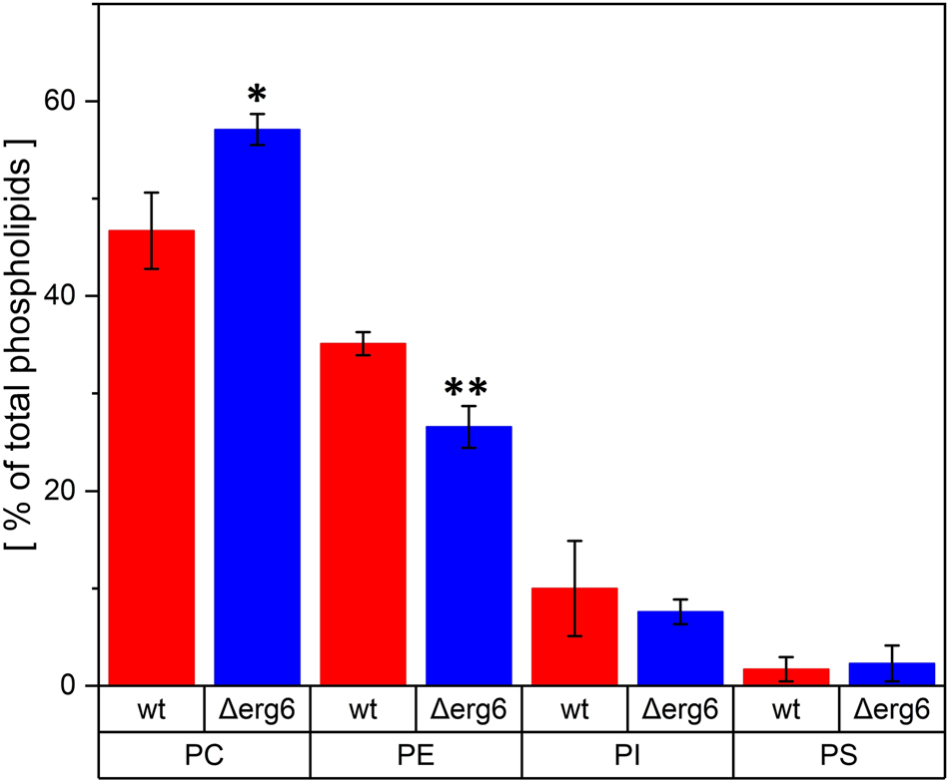
Content of selected phospholipids (phosphatidylcholine (PC), phosphatidylethanolamine (PE), phosphatidylserine (PS), and phosphatidylinositol (PI)) in membranes of *C. glabrata*. (*p = 0.0027; **p = 0.0005; unpaired t-test *Δerg6* versus *wt*; results are expressed as mean values of four independent experiments ± standard deviations)

When analysing the phospholipid profile of *C. glabrata* membranes, our focus was on the four primary phospholipids present in the membranes: phosphatidylcholine (PC), phosphatidylethanolamine (PE), phosphatidylserine (PS), and phosphatidylinositol (PI). Differences were observed in all phospholipids analysed across the strains (Fig. 2), but only the differences in PC and PE content were statistically significant. *Δerg6* cells contain higher amounts of PC and lower amounts of PE compared to the *wt* strain (Table 2). The total amount of analysed phospholipids was almost identical in both samples (*wt* = 3.01 ± 0.43 µg and *Δerg6* = 3.18 ± 0.61 µg).

**Table 2:** Content of phosphatidylcholine (PC), phosphatidylethanolamine (PE), phosphatidylinositol (PI) and phosphatidylserine (PS) in the membranes of *C. glabrata*

	*wt [%]*	*Δerg6 [%]*	p-value
**PC**	46.70 ± 1.96	57.10 ± 0.80	0.0027
**PE**	35.09 ± 0.59	26.59 ± 1.07	0.0005
**PI**	10.00 ± 2.44	7.60 ± 0.63	0.3700
**PS**	1.70 ± 0.61	2.30 ± 0.91	0.5885

% of total phospholipid content in the samples

The results are expressed as mean values of four independent experiments ± standard deviations

### Difference in Transmembrane Potential of the *C. glabrata*

**Fig. 3:**
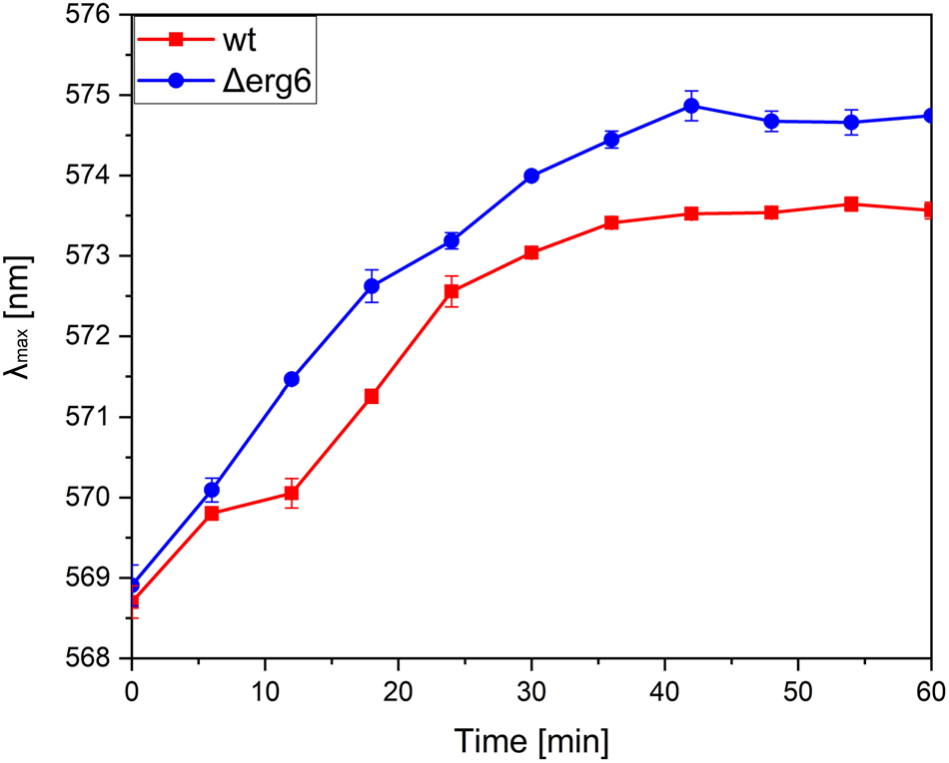
Average changes in the emission maximum position of the diS-C3[3] probe after incubation in a suspension of *C. glabrata*. (p < 0.05; unpaired t-test *Δerg6* versus *wt* at 60 min; results are expressed as mean values of three independent experiments ± standard deviations)

The transmembrane potential (TMP) in the studied *C. glabrata* strains was quantified using the diS-C3 [3] probe (Fig. 3). Based on the λ_max_ fluorescence of the diS-C3[3] probe in its saturated state after 60 min of incubation in *C. glabrata* cells, where the emission maximum shifted from 569 nm to 575 nm for *Δerg6* compared to a shift from 569 nm to 573 nm for *wt*, we calculated the TMP difference between *Δerg6* and *wt*. The cytoplasmic membrane of *Δerg6* hyperpolarized by 6.94 ± 0.31 mV more than the wt strain.

### Susceptibility of *C. glabrata* to Metabolic Inhibitors

**Fig. 4:**
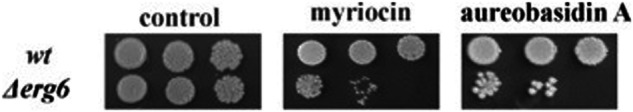
Growth of *wt* and *Δerg6* mutant in the presence of myriocin and aureobasidin A. 5 µl aliquots of tenfold serial dilutions (10^7^, 10^6^, 10^5^ cells/ml) of overnight cultures were spotted onto YPD plates and incubated at 30 °C for 2 days

The *Δerg6* strain was found to be more sensitive to myriocin (a serine-palmitoyltransferase inhibitor) and aureobasidin A (an inositol phosphorylceramide (IPC) synthase inhibitor) compared to the wt strain (Fig. 4).

### *PDR16* Gene Expression in *C. glabrata* after *ERG6* Gene Deletion

**Fig. 5:**
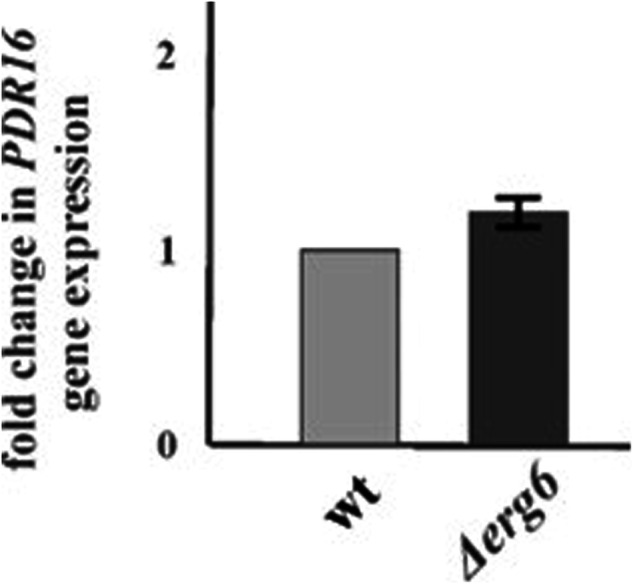
Relative gene expression levels of *PDR16* in *C. glabrata Δerg6* deletion mutant. The gene transcript levels in *wt* were set as 1. (p = 0.117; unpaired t-test *Δerg6* versus *wt*; results are expressed as mean values of three independent experiments (± standard deviations) normalized to the *C. glabrata ACT1* gene expression level)

To understand how sterols affect the cellular distribution of phosphatidylinositol and the relationship between the *ERG6* and *PDR16* genes in *C. glabrata*, we monitored the expression of the *PDR16* gene in the *Δerg6* strain. We did not observe any changes in the expression of the *PDR16* gene (Fig. 5).

## Discussion

Sterols are lipids essential for many cellular processes, and even small changes in their structure or composition can significantly impact cell physiology. The sterol profile is crucial for cells under stress conditions [41]. Deletion of genes involved in the ergosterol biosynthetic pathway strongly affects the nature of sterols accumulated by yeast cells. The ERG6 gene encodes a protein responsible for the methylation of zymosterol at C-24 [42].

Sterol profiling in *S. cerevisiae* has shown that *ERG* genes have reduced substrate specificity and can process a wide range of similar sterol structures. As a result, mutants of late *ERG* genes accumulate mixtures of sterols, including the precursors they lack [43, 44]. Small changes in the sterol methyl groups and the positions of unsaturation in the ring structure or the aliphatic tail of the molecule significantly affect the biophysical properties of the plasma membrane in *S. cerevisiae* [45, 46] These findings have significant implications for understanding the mechanism of action of various antifungals that specifically target ergosterol biosynthesis. The efficacy of polyenes on clinical isolates of *C. glabrata* with a mutation in the *ERG6* gene has been found to be reduced [20], as ergosterol is the primary target molecule for their action, explaining the reduced efficacy in the absence of the ergosterol-producing gene. Deletion of the *ERG6* gene leads to increased tolerance to azole antifungals in *C. glabrata* [47]. Deletion of the *ERG3* or *ERG11* genes results in increased susceptibility to azole antifungals in *C. glabrata*, but the simultaneous deletion of both genes makes cells resistant not only to azole antifungals but also to amphotericin B [10]. In *C. glabrata* cells exposed to azole antifungals, increased activity of the *ERG2*, *ERG4*, and *ERG10* genes is believed to contribute to defence against their action [48].

Ergosterol is an essential component of fungal cell membranes [49]. Its deficiency leads to changes in the standard properties of membranes and affects various biological processes [50]. In our experiments, we observed ergosterol depletion in *Δerg6* deletion mutant (Fig. 1, Table 1), consistent with the findings of [20, 51]. In the yeast *K. lactis*, ergosterol levels are undetectable upon deletion of the *ERG6* gene [17]. Our experiments recorded increased levels of zymosterol in the membranes of the *Δerg6* strain compared to *wt* as a consequence of *ERG6* deletion. The accumulation of zymosterol in cells due to *ERG6* gene deletion has been observed in studies on *C. albicans* [52], *S. cerevisiae* [53], *K. lactis* [17], as well as in various *Aspergillus yeast* species [22].

**Fig. 6:**
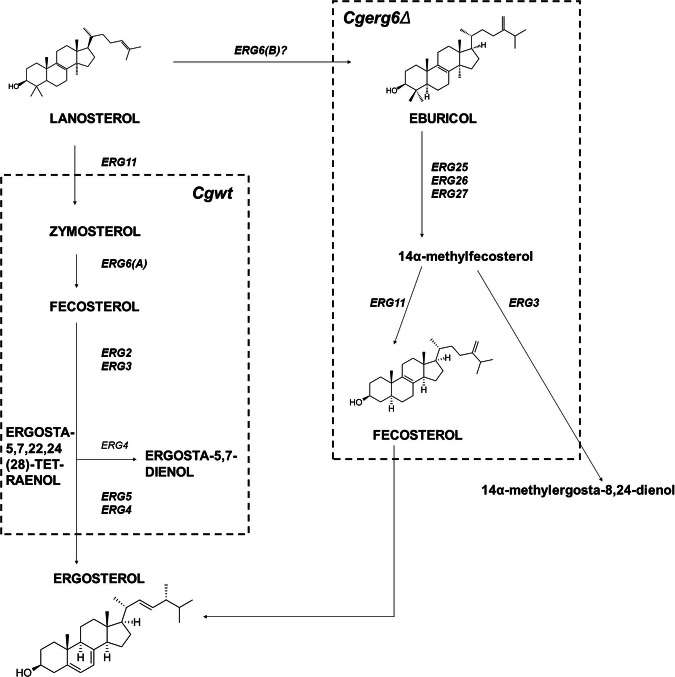
Proposed alternative pathway for ergosterol synthesis using eburicol in *C. glabrata Δerg6*

Ergosterol levels within *Δerg6* presented in this study are very low (Fig. 1, Table 1), but it´s presence in *Δerg6* can be explained by its synthesis via eburicol [54]. Our results demonstrate a significant increase in the amount of eburicol in the *Δerg6* strain compared to the *wt* strain, suggesting possible ergosterol synthesis through eburicol. This synthetic pathway for ergosterol is known in the yeasts *Aspergillus fumigatus* [55] and *Cryptococcus neoformans* [9]. Although this synthetic pathway requires an active *ERG6* gene for eburicol synthesis, the study by [55] indicates that *Candida* yeast have developed an analogous synthetic pathway that allows ergosterol synthesis from other intermediates, but it does not explain the presence of ergosterol in cells with a deleted *ERG6* gene. The possibility of ergosterol synthesis via eburicol has not yet been studied in *C. glabrata*. Therefore, we propose an alternative pathway for ergosterol synthesis (Fig. 6), which may explain its presence in the *Δerg6* strain. Other potential explanations for the presence of ergosterol in *Δerg6* cells may include the variability of the entire *C. glabrata* genome and possible activity of methyltransferases that might partially compensate for the missing 24-C-methyltransferase. In *Trypanosoma brucei*, a new gene has been discovered that can catalyse the conversion of zymosterol to fecosterol without the presence of 24-C-methyltransferase [56]. We cannot rule out the possibility of ergosterol synthesis in *Δerg6* through this pathway, as a complete genome analysis on methyltransferases has not yet been conducted.

Phospholipids form the main structural component of all membranes, and their composition is influenced by sterols in the membrane [57]. In *Δerg6*, we observed a significantly increased PC content and a decreased PE content compared to *wt*, but no change in the levels of PS and PI. PC is the most abundant eukaryotic phospholipid, important for membrane structure due to its cylindrical shape [27]. PE primarily serves as a precursor for PC synthesis and as a substrate for important post-translational modifications [58]. In most yeast membranes, the PC and PE content ranges from 60 to 70% of the total phospholipids [27]. However, in the *C. glabrata* strains we observed, this content reaches higher values, up to 81.79% in wt and 82.69% in *Δerg6*, corresponding to the values of model membranes of filamentous fungi [59]. These values are consistent with the results of [60], where the PC and PE content was 78.125% in a standard *C. glabrata* strain. The PC to PE ratio (PC:PE) affects yeast membrane fluidity, integrity, and stability, lipid biosynthesis and transport [27], stress adaptation, membrane protein function [61], membrane permeability [62], yeast pathogenicity [25], and antifungal efficacy [63]. Maintaining the correct PC:PE ratio is therefore crucial for the health and functioning of yeast cells, influencing their ability to adapt to various environmental conditions and perform essential cellular processes. While the PC:PE ratio is 1.33 in *wt*, it reaches 2.14 in *Δerg6*, confirming that along with the lack of ergosterol, there is a reduction in membrane fluidity in the *Δerg6* mutant compared to *wt* [47]. A decrease in the PC:PE ratio leads to higher efficacy of azole antifungals in *C. albicans*, where a clinical isolate with a lower ratio showed higher sensitivity to these substances than those with a higher PC:PE ratio [63]. However, this conclusion contradicts our previous results on the efficacy of azole antifungals on *Δerg6 C. glabrata* [47], as the increase in the PC:PE ratio did not indicate increased sensitivity of the *Δerg6* mutant to azole antifungals. In S. cerevisiae, deletion of the *PDR16* and *PDR17* genes, which regulate levels of various lipids, leads to hypersensitivity to azole antifungals [36]. Reduced PE content in sensitive *C. albicans* strains induces lower activity of efflux transporters and thus higher efficacy of azole antifungals [64]. The results for PS and PI content align with the effects of PS and PI on membranes, where membranes with higher PS content were significantly more condensed and organized, while an increase in PI content increased their fluidity [59].

Myriocin is a potent inhibitor of serine-palmitoyltransferase, the first step in sphingosine biosynthesis, affecting lipid composition in the plasma membrane and the biophysical properties of the membrane. Aureobasidin A is a unique yeast antibiotic that kills *S. cerevisiae* even at low concentrations by inhibiting inositol phosphoryl ceramide synthase, an essential enzyme in yeasts. [46] demonstrated the interaction between sterols and sphingolipids in yeast cells. Cells respond to sterol structures by altering the composition of their membranes, leading to preferential modifications of sphingolipids. The altered sterol composition in the *Δerg6* strain induces a specific sphingolipid pattern, which is related to the observed sensitivity to myriocin and Aureobasidin A, specific inhibitors of sphingolipid biosynthesis. Our results also indicate different interactions between ergosterol metabolism and lipid transport within yeast cells of *K. lactis* and *C. glabrata*. The lipid transfer protein Pdr16p compensated for the effect of the *ERG6* gene mutation in *K. lactis* [17], but this phenomenon was not confirmed in the studied *C. glabrata* strain.

We observed that the deletion of the *ERG6* gene induces hyperpolarization of the plasma membrane in the *Δerg6* yeast strain by 6.94 ± 0.31 mV compared to the wt strain. This result aligns with the findings of [47]. Changes in the sterol component of plasma membranes also cause hyperpolarization of the plasma membrane in *S. cerevisiae* [65]. In *Candida* species, it has been demonstrated that hyperpolarization of the plasma membrane leads to increased penetration of azole antifungals into the cells [66], ultimately increasing their efficacy and leading to higher cell mortality [65, 67]. The main mechanism of protection for *C. glabrata* cells against the effects of azole antifungals is the increased activity of efflux ABC pumps [68, 69]. The exact value of the resting TMP in *C. glabrata* has not yet been determined because the small size of the cells [70] makes it difficult to measure accurately using available experimental techniques. We can only estimate its value to be similar to that of other *Candida* yeasts. *C. albicans* cells have a resting TMP value of 120 mV [71].

## Conclusion

We have demonstrated that the disruption of ergosterol biosynthesis impacts the overall lipid composition in membranes, causing the accumulation of zymosterol, depletion of ergosterol, and hyperpolarization of the plasma membrane *Δerg6* strain. We also found that despite the blockade of the ergosterol biosynthetic pathway, we can still observe low levels of ergosterol. The presence of ergosterol can be observed in *Δerg6* cells, suggesting the existence of an alternative pathway for its synthesis, which may utilize eburicol for its function.

## Data Availability

No datasets were generated or analysed during the current study.
